# Communicative Profiles of Children with Developmental Delay Compared to Age- or Language-Matched Typically Developing Peers

**DOI:** 10.1007/s10803-025-06882-0

**Published:** 2025-05-23

**Authors:** Ronit Saban-Bezalel

**Affiliations:** Department of Communication Disorders, Ariel University, Ariel, Israel

**Keywords:** Pragmatics, Communicative intentions, Means of communication, Developmental delays

## Abstract

Expressing communicative intentions is a fundamental pragmatic skill that develops early in childhood, enabling individuals to meet personal and social needs. This ability relies on cognitive, social, and linguistic competencies, which can pose challenges for children with intellectual disability (ID) or global developmental delays (GDD). This research examines the communicative profile, including the communicative intentions produced and the means of production of young children with ID or GDD (the developmental delay group-DD) compared to their typically developing (TD) peers during a structured pragmatic protocol. Seventy-two children aged 24 to 68 months participated in the study, divided across three groups: twenty-three children with DD, twenty-three TD children matched by chronological age to the children with DD, and twenty-five TD children matched by language age to the children with DD. All children underwent a structured pragmatic observation protocol that prompted the production of communicative intentions. Significant differences were observed when comparing children with DD to their age-matched TD peers, namely, TD children produced more social communicative intentions and used more complex means of production. A more similar communicative profile was found between the DD group and equivalent language-matched TD peers. Language ability was associated with producing a greater variety of communicative intents for both language-matched groups; however, better executive functions were associated with a broader variety only among younger TD children. This study highlights delays in communicative profiles and differences in related variables between children with DD and their TD peers of equivalent chronological age, and of equivalent language age.

**Supplementary Information:**

The online version contains supplementary material available at https://doi.org/10.1007/s10803-025-06882-0.

## Introduction

Intellectual disability (ID) and global developmental delay (GDD) are neurodevelopmental disorders diagnosed during the developmental period. ID involves intellectual and adaptive functioning deficits across conceptual, social, and practical domains (DSM-5, 2013). Significant delays in multiple developmental domains characterize GDD and are specifically identified in children under five who cannot be reliably assessed using standard intellectual measures (DSM-5, 2013; Shevell, 2008). Studies indicate that children initially diagnosed with GDD often continue to demonstrate considerable developmental challenges as they age (Lee et al., 2022; Shevell et al., 2005a, 2005b). Given the overlapping characteristics of these conditions, including impaired cognitive, motor, language, and social skills, it is justifiable to group them in research (Pinchefsky & Shevell, 2017; Shevell, 2008). The current study explores communicative intent development among children with ID or GDD.

### Pragmatic Language

Pragmatic language refers to the ability to use language appropriately in social contexts (Airenti, 2017). It begins developing in infancy, starting from the preverbal stage when infants engage in dyadic interactions, including joint attention and gestures (Airenti, 2017). These early interactions help children assign meaning and establish references, allowing them to learn from others’ language (Clark, 2014) as well as acquire essential social skills that enable them to navigate the social world (Airenti, 2017; Bohn & Frank, 2019) and significantly influence their overall quality of life (Nota et al., 2007). Pragmatic language encompasses three primary areas: (1) communicative intentions, such as requesting or commenting, which can be direct or indirect; (2) presupposition, which involves gauging the listener’s knowledge needs, information processing abilities, and social status to adjust communication; and (3) discourse management, which includes initiating, maintaining, and concluding conversations appropriately (Bates, 1976; Landa, 2005; Rivers et al., 2012). The current study will focus on the first area of pragmatic language- communicative intents.

### Communicative Intentions Development

Joint attention is a fundamental milestone in early social-cognitive development, emerging around 9 to 12 months of age. It marks the transition from dyadic interactions to triadic interactions, where an infant, an adult, and an external entity coordinate attention. This shift signifies the infant’s growing understanding of themselves and others as intentional agents—individuals directing attention and actions toward goals. Building on this foundation, infants soon develop an understanding of communicative intentions, a special form of intentionality in which they recognize that others perceive and act upon the world and intend to influence their attentional states. Recognizing that someone else wants them to attend something is a prerequisite for understanding communicative intentions, forming the basis for social communication and language development (Tomasello, 2024).

Expressing communicative intentions is a fundamental pragmatic skill that develops early in childhood and enables individuals to fulfill their personal and social needs (Abbeduto et al., 1988). These intentions, when vocalized, are referred to as speech acts (Adams, 2002). The speaker articulates their intent through these speech acts, influencing the listener in specific contexts. Communicative intentions consist of two key components: 1) the means of communication, such as gestures, vocalizations, or speech, and 2) the intended function or meaning (i.e., request, protest, or comment) (Calandrella & Wilcox, 2000). Typically, these communicative goals are classified as either declarative, serving social purposes, or regulatory, aimed at requesting or controlling an interaction for non-social objectives (Landa, 2005). In daily interactions, expressing and responding to speech acts is essential for active participation in communication. Those who excel in this area engage more fully in social exchanges, while those who struggle may miss crucial social and linguistic experiences (Abbeduto et al., 1988).

In typically developing infants (TD), early communicative intents, such as attracting attention, requesting actions or objects, and naming, emerge primarily through non-verbal means like pointing, hand extensions, vocalizations, gestures, and eye contact (Abbeduto & Hesketh, 1997), or intonation (Zhang et al., 2024). Around 12 months, these expressions become integrated with speech and include declarations, requests, expressions of needs, demands for action, demands for an answer, protests, answers to questions, and responses to interactions. Rapid communication skill progression occurs between 14 and 32 months, with basic speech acts generally mastered by ages 3 to 4 (Klecan-Aker & Swank, 1988; Nikolaus et al., 2022; Snow et al., 1996; Sylvestre et al., 2023). Other speech acts, such as clarification, are acquired beyond four to five years (Klecan-Aker & Swank, 1988), and more complex speech acts like promises, persuasion, polite forms, and justifications develop later in age continuing into adolescence (Adams, 2002; Nikolaus et al., 2022). Recently, Sylvestre et al. (2023) examined the developmental trajectory of communicative intentions in preschool children aged 3 to 4.5 years within a play-based context. Their findings align with previous studies, showing a significant increase in using communicative intents with age. At age three, at least 50% of the children demonstrated the following intents: suggesting, protesting, reporting an event, sharing preferences, accompanying actions, announcing actions, and explaining. By 4.5 years, the production of these intents increased to 90–100%. Notably, arguing was the only intent produced by fewer than half of the children at 36 months. Over time, children expand their range of communicative intentions and improve communication clarity and effectiveness (Snow et al., 1996). Nikolaus et al. (2022) confirmed previous findings on speech act development using a large-scale automatic annotation model. Specifically, the model was trained and validated on a dataset originally annotated by Snow et al. (1996). The model successfully replicated key findings, including the developmental trajectory of speech acts and individual variations among children. Demonstrating that communicative intents follow a similar trajectory in a broader dataset.

### Foundations of Pragmatic Abilities

The Emergentist Model of Pragmatic Ability and Disability (Perkins, 1998, 2000, 2005; Perkins, & Azios, 2024) views pragmatics as an emergent phenomenon rather than a discrete communicative component like syntax or phonology. This model, rooted in the interactionist approach, suggests that pragmatic ability arises from the dynamic interplay of various cognitive, linguistic, and sensorimotor systems. When individuals communicate, they rely on multiple interacting capacities, including (1) language, gesture, and facial expression, (2) cognitive functions such as theory of mind, inference, executive function, and memory, (3) motor output systems like speech articulation and hand movement, and (4) sensory input systems such as hearing and vision. Importantly, this model highlights how pragmatic behavior may often result from compensatory adaptations, where deficits in one system may be offset by reliance on another. From a clinical perspective, the emergentist approach provides a more comprehensive framework for understanding pragmatic difficulties, allowing clinicians to target underlying causes. This perspective shifts the focus from viewing pragmatics as a fixed skill to recognizing it as a dynamic process shaped by an individual’s communicative resources and the demands of interaction (Perkins, & Azios, 2024). The model is clinically based on a wide range of development (e.g. ASD, SLI) and acquired language pathologies (Aphasia) (Perkins, 1998, 2000, 2005). Thus, difficulties in understanding speech acts or managing conversational turns can arise from challenges within these underlying systems. Indeed, research has demonstrated links between pragmatic skills and broader developmental capacities like cognitive abilities such as executive function abilities, theory of mind, and structural language (Blain-Brière et al., 2014; Lee et al., 2017; Gooch et al., 2016; Matthews et al., 2018).

### Executive Functions and Communicative Intents

Executive functions are cognitive skills that support goal-directed behavior and self-regulation, including working memory, inhibition, cognitive flexibility, attention, and planning, which begin developing in early childhood (Garon et al., 2008). These cognitive processes help young children regulate their behavior and lay the foundation for adaptive, goal-directed actions, overriding automatic responses (e.g., screaming, crying), or impulsive behaviors (e.g., grabbing or throwing objects). As such, executive functions play a critical role in learning and contribute to developing social, emotional, and cognitive competence (Griffin et al., 2016). They are also essential for daily interactions and pragmatic language use (Lee et al., 2017). Indeed, previous research has demonstrated a relationship between executive functions and pragmatic abilities in both TD children (Blain-Brière et al., 2014; Matthews et al., 2018) and school-age children with intellectual disabilities (Lee et al., 2017).

Conveying and responding to communicative intentions rely on several executive processes. Working memory enables individuals to retain and process linguistic information, while inhibition allows speakers to suppress their perspectives when necessary. Cognitive flexibility supports the ability to adapt responses to fit the communicative partner’s utterances, and attention is essential for comprehending utterances and integrating contextual information. Additionally, planning helps anticipate and formulate the next communicative intention (Cummings, 2023; Matthews et al., 2018). Deficits in one or more of these abilities can shape the trajectory of pragmatic development and may explain deviations from typical language acquisition in children with developmental disorders (Matthews et al., 2018).

Since pragmatic competence relies on cognitive skills, social knowledge, and linguistic abilities (Matthews et al., 2018), individuals with ID or GDD—who often face challenges in these areas—may experience difficulty in effectively expressing and interpreting communicative intentions.

### Difficulties in Pragmatic Abilities and Communicative Intentions Among Individuals with ID and GDD

Notable difficulties in pragmatic abilities are evident in individuals with ID from unknown causes as well as those with specific genetic syndromes such as Down syndrome (DS), Fragile X, and Williams syndrome (Martin et al., 2017). These difficulties manifest across various pragmatic functions, including elaborating on topics, repairing communication breakdowns, initiating conversations, telling cohesive narratives, turn-taking, establishing referents, maintaining topic relevance, performing speech acts, and interpreting paralinguistic cues. These challenges affect both expressive and receptive communication and persist into adulthood (Abbeduto, 1991; Abbeduto & Hesketh, 1997; Djordjevic et al., 2023; Martin et al., 2017).

Several studies have explored the broad pragmatic difficulties faced by individuals with ID. For example, Smith et al. (2017) used the Children’s Communication Checklist (CCC; Bishop, 2003a, 2003b) to compare the pragmatic abilities of six-year-olds with DS to age-matched TD peers, finding significantly weaker pragmatic skills in the DS group, even when matched by mental age. Despite strengths in non-verbal communication, children with DS struggled with peer conversations. Lee et al. (2017) extended this research using the CCC-2 and conversational assessments, confirming that pragmatic difficulties persisted even after adjusting for mental age and language ability. Moreover, theory of mind and executive functions were found to correlate positively with pragmatic competence.

While these studies emphasize the broad scope of pragmatic challenges in individuals with ID, research specifically examining communicative intentions in this population remains scarce. Abbeduto (1991) reviewed evidence suggesting that individuals with ID exhibit delays in speech acts, such as using polite indirect speech and comprehending others’ speech acts. However, he noted that older individuals with ID can perform speech acts effectively in familiar situations with minimal cognitive and linguistic demands. Yoder and Warren (2002) further found that toddlers with DS struggled to acquire and produce communicative intentions, particularly requests. Similarly, Coggins et al. (1983) observed that the variety of communicative intentions expressed by children with DS at a two-word developmental level was comparable to that of TD children matched by cognitive and linguistic abilities.

Research on the pragmatic abilities of children with GDD is also limited. Existing studies highlight significant challenges in social-communicative skills, such as difficulties with gestures and triadic gaze coordination (Veness et al., 2014; Ventola et al., 2007). Delehanty et al. (2018) examined social-communicative abilities in children across five neurodevelopmental disorder subgroups (including autism and developmental delays) compared to TD children using the Communication and Symbolic Behavior Scales (CSBS) (Wetherby & Prizant, 2003). Their findings revealed significant differences between TD children and all other subgroups. Children with GDD exhibiting social challenges similar to those observed in children with autism—particularly in emotional expression, eye contact, and gesture use. These findings highlight the difficulty in distinguishing GDD from autism in social-communicative contexts (Veness et al., 2014; Ventola et al., 2007).

Further, studies examining communicative development in young children with significant cognitive and motor delays (ages 6 to 59 months) have shown that these children exhibit delays in acquiring communicative functions compared to TD children aged 0–24 months. Specifically, they tend to communicate at the level of pre-intentional and intentional behaviors, with communicative acts primarily serving functions such as refusing (i.e., protesting), obtaining (i.e., continuing an action), and, to a lesser extent, social engagement (i.e., attracting attention). These intentions are expressed through early sounds, body movements, and facial expressions. Additionally, a strong relationship has been observed between communicative ability and motor functioning in this population (Dhondt et al., 2020).

While existing studies provide valuable insights into early communication in children with ID and GDD, research specifically examining the range and nature of communicative intentions in these populations remains scarce. Given their foundational role in pragmatic development and everyday functioning, understanding how children with ID and GDD express and produce communicative intents is crucial.

To address this gap, the present study systematically investigates communicative intentions as a key aspect of pragmatic language in preschool-aged children with ID or GDD. By comparing their performance to that of TD peers matched by chronological age and TD peers matched by language ability, we aim to gain deeper insight into their communicative abilities and the underlying processes that support their acquisition. Comparing children with ID or GDD to both age-matched and language-developmentally matched peers is essential for distinguishing whether differences in communicative intentions result from overall developmental delay or are influenced by other cognitive and social factors shaping pragmatic development. This dual comparison provides a more comprehensive perspective and helps characterize the communicative abilities of children with ID or GDD in early development. Specifically, we examine the quantity and variety of communicative intentions produced and the means used to express them, collectively referred to as the *communicative profile*.

### Current Study Objectives

This study has two primary objectives:

1. To compare the *communicative profile*, including the variety of communicative intentions (number of different communicative intentions produced), the quantity (i.e., sum) of each communicative intention produced, and the means used to express these intentions—between children with ID or GDD and TD children matched by language age and TD children matched by chronological age during a structured pragmatic observation protocol.
2. To examine the relationship between each group’s characteristics (age, cognitive functioning, language abilities, executive functions, and adaptive behavior) and the variety of communicative intentions they produce during the structured pragmatic observation protocol.

## Method

### Participants

Seventy-two children aged 24–68 months were recruited for the study. They were divided into three groups: the study group, which included children with ID, GDD, or DS, and two control groups, one consisting of TD children matched chronologically to the study group and the other comprising TD children matched based on language abilities.

Inclusion criteria for the study group clinically diagnosed children in the monolingual phase of language acquisition at a minimum (i.e., producing at least one word), with cognitive function at least two standard deviations below the average (< 70). Cognitive functioning was examined using the Mullen Scales of Early Learning (MSEL) (Mullen, 1995). The group included 23 children (9 girls) aged 43–68 months (M = 55.17, SD = 5.47). Among this group, prior to the study, children have been clinically diagnosed based on multidisciplinary assessments conducted by certified professionals (e.g., developmental pediatricians, psychologists, or speech-language pathologists). Children were diagnosed with GDD (n = 14), moderately severe ID (n = 1), moderately severe ID and cerebral palsy (n = 1), mildly severe ID (n = 1), and DS (n = 6). All the children in the study group attended specialized educational programs designed for children with developmental delays or intellectual disabilities. Within these settings, they received tailored intervention programs aligned with their academic plans and health-related therapies. The study group will be referred to as the *Developmental Delay group* (DD).

Since pragmatics is a key component of linguistic ability, and both expressive and receptive language are closely linked to pragmatic skills (Matthews et al., 2018), the first control group consisted of young TD children matched to the study group (DD group) based on age equivalence in expressive and receptive language. This equivalence was determined using the MSEL(Mullen, 1995), [*F*(2,48) = 1.77, *p* = 1.8, Wilk’s Λ = 0.08, η2 = 0.7]. This group consisted of 25 children (14 girls) aged 24–42 months (M = 31.32, SD = 4.95) and is referred to as the *typically developed age-equivalent language group (TD-LA).*

The second control group consisted of 23 TD children (8 girls) aged 42–68 months (M = 55.39, SD = 6.83), who were group-matched by chronological age to the study group. This group is referred to as the* typically developed chronological age-matched group (TD-CA).*

Chi-square analysis indicated that the three groups did not differ in gender distribution, χ^2^(2) = 2.65, *p* = 0.265. All the children in the study were Caucasians and of Jewish ancestry. They were all native Hebrew speakers. Other demographic features and background measures are summarized in Table 1.

**Table 1:** Descriptive statistics: mean, SD and ANOVA F values of the children’s characteristics by group

	Study group								
	1 GDD (*n* = 23)		2 TD-LA (*n* = 25)		3 TD-CA (*n* = 23)		F values		
	Mean	SD	Mean	SD	Mean	SD	*F*	*η*_*p*_^2^	Scheffe
*Background measures*									
Children's age in months	59.30	8.96	31.32	4.95	55.39	6.83	112.16***	.76	2 < 1 = 3
MSEL T score	55.17	5.47	103.36	15.30	100.39	10.87	130.62***	.79	1 < 2 = 3
Receptive language age equivalence	29.75	6.32	31.40	6.03	52.83	7.70	97.86***	.74	1 = 2 < 3
Expressive language age equivalence	27.08	7.77	30.88	4.88	54.60	9.97	85.87***	.72	1 = 2 < 3
VABS—total	63.26	10.19	101.72	10.02	105.17	12.06	108.40***	.76	1 < 2 = 3
Brief-P (EF) standard score	68.22	14.11	47.36	6.52	47.35	10.57	29.79***	.47	2 = 3 < 1

*GDD* global developmental delay, *TD-LA* typically developed age-equivalent language, *TD-CA* typically-developed- chronological age, *MSLE* Mullen Scales of Early Learning (MSEL; Mullen, 1995); *VABS* Vineland Adaptive Behavior Scales (Sparrow et al., 2005), *EF* executive function

***p <.001

### Procedure

The University Ethics Committee and the Ministry of Education Ethics Committee approved the study. Parents of children with DD were approached through special education preschools, and parents of TD children were approached by personal acquaintances. After parents consented to their child’s participation in the study, they filled out a demographic questionnaire and participated in a parental interview regarding their child’s adaptive functioning. The children’s preschool teacher completed a questionnaire on the child’s executive functions (Brief-P measure). All children participated in one or two sessions with a researcher, during which they underwent a developmental assessment and engaged in the structured pragmatic observation protocol. The researcher, a speech and language pathologist with extensive clinical experience working with children with developmental delays (the author) administered the pragmatic protocol to the study group and trained the research assistants to administer it to the TD children. All trained research assistants were undergraduate students studying Communication Disorders and had experience working with young children.

### Measures

#### Mullen Scales of Early Learning (MSEL; Mullen, 1995)

The MSEL assessment measures development in infants and preschoolers from birth to 68 months. The test yields five standard sub-scale scores, including gross motor functions, visual reception, fine motor skills, and receptive and expressive language. The test also yields an early learning composite score representing overall cognitive functioning (mean = 100; SD = 15) based on T scores of four cognitive sub-scale scores (visual reception, fine motor skills, and receptive and expressive language). The scoring range for each test item varies from zero to five points. The Mullens composite score has high internal reliability with a median value of 0.91. Mullens composite score correlated 0.70 with the Bayley Mental Developmental Index supporting the validity of the Mullen Early Learning Composite score as a measure of global cognitive ability.

#### Vineland Adaptive Behavior Scales, Second Edition, (VABS; Sparrow et al., 2005)

This standardized caregiver interview was designed to assess adaptive behaviors in children from birth to 18 years of age. Its four subdomains (communication, daily living skills, socialization, and motor skills) each yield a standard score. Additionally, this instrument yields an adaptive behavior composite total score (M = 100; SD = 15). Higher scores reflect better functioning. The VABS has a high internal consistency reliability of 0.90–0.80 and a high validity of > 0.90 in all age groups except adolescents 83.

#### Behavior Rating Inventory of Executive Function Preschool (Brief-P; Sherman & Brooks, 2010)

The questionnaire is a standardized rating scale for children aged 2–5:11 years, designed to specifically measure executive function in preschool-aged children by parents and teachers of preschool-aged children; thus, it enables the assessment of executive function behaviors in the home and preschool environments. It contains 63 items within five nonoverlapping clinical scales measuring executive functioning: Inhibit, Shift, Emotional Control, Working Memory, and Plan/Organize. Internal consistency reliability for these scales ranges from 0.80 (Plan/Organize) to 0.90 (Inhibit). From the scales, the clinicians derive three broader indices: inhibitory self-control, flexibility, and emergent metacognition, and a general composite score can also be calculated. Brief -P has a high internal consistency reliability of 0.80 −0.97.and and a high validity of 0.88–0.90.

#### Structured Pragmatic Observation Protocol

The pragmatic protocol conducted was based on the ’Peanut Butter Protocol’ (Creaghead, 1984), a non-standardized protocol for eliciting pragmatic behaviors from preschool children (ages 3–5) in a ‘communicative demand’ activity. We used the *Drawing* format with minor modifications, focusing only on the first part of the protocol, which examines communicative intents. During the observation, the examiner follows a protocol that prompts the child to express communicative intentions naturally. The protocol elicited the following communicative intentions: Deny or object, request an object or action, make choices, comment, request clarification, request information, guess, greet, answer ‘wh’ questions, draw attention, and conversation. The administration of the protocol to all the children in the study took approximately 15 min. See Appendix A.

##### Coding and Analysis

The children’s performance was coded if after being prompted, they produced the designated communicative intent; if they produced after the prompt, any of the other communicative intentions aimed to be elicited during the protocol (i.e., instead of asking for the box, the child asked, “What is in the box?”); if they spontaneously produced one of the communicative intents aimed to be elicited during the protocol regarding any of the protocol tasks (i.e., While the child was drawing, they commented, “I am drawing a rainbow.”). Children’s performance was coded as engaging in a conversation with the examiner if they expanded on the examiner’s attempt to initiate a conversation (e.g., by adding information or asking a question). For each prompt in the protocol, a binary coding system was used (1 = communicative intent produced; 0 = not produced), based on the child’s response.

In addition, the means used (verbal or non-verbal) to produce each of the communication intents (i.e., body action, words, gestures) were also coded. If a child coordinated vocalization and gesture in their intent, it was coded under both vocalization and gesture. The pragmatic observation was recorded using a video camera and later analyzed using the Noldus Observer software (Wageningen, Netherlands).

Based on the protocol analysis, a communicative profile was coded for each child. The profile included three components: 1. Frequency of occurrence for each communicative intent—the total number of times each communicative intent was produced.2. Variety of communicative intents—the number of different communicative intents used by each child, regardless of frequency.3. Frequency of communicative means—the number of times different communicative means were used.

##### Reliability and Examiner Training

The author coded all the protocols. Initially, the author and a research assistant independently coded the recorded protocols. After establishing consensus regarding the coding procedure, inter-rater reliability was evaluated. For this purpose, 20% of the children’s protocols were randomly selected and independently coded by the author and a different trained research assistant. Cohen’s kappa, calculated using statistical software, yielded a value of 0.81 for identifying communicative intents and their modes of production, indicating strong inter-rater agreement.

All examiners who conducted the protocol were third- or fourth-year students specializing in communication disorders. They had completed their clinical practicum and were familiar with various communication disorders. Before administering the protocol, all examiners underwent comprehensive training conducted by the author. The training included: 1. Demonstration of the protocol; 2. Video review of a recorded session illustrating the procedure, 3. Practical implementation, each examiner conducted the protocol with one child and then reviewed the recording with the author to verify adherence to the protocol and ensure consistency across examiners. To ensure procedural fidelity, the researcher administering the pragmatic protocol had access to detailed procedural guidelines throughout the session.

## Data Analysis

First, we compared several child characteristics across the three groups using one-way univariate analyses of variance (ANOVA). These characteristics included age, cognitive functioning, receptive and expressive language equivalent age, adaptive functioning, and executive function. Since a normal distribution was not observed for the dependent variables, non-parametric tests were performed to avoid parametric biases. For our first research question Planned comparisons (k-1) were conducted to examine differences between the study group and each control group, following a priori determinations based on the study’s aims. Mann–Whitney U tests were used to compare scores between the study group and each control group (independent variable) on the following dependent variables: Frequency of communicative intentions; Variety of communicative intentions; and means of expression. For our second research question, Spearman correlations were calculated separately for each group to examine the associations between the variety of communicative intentions produced and various personal variables, including age, cognitive functioning, language abilities, executive functions, and adaptive behavior. To compare the correlation coefficients between groups, we conducted a non-parametric permutation test (Good, 2000). This test is preferred for small samples because it does not assume normality and directly evaluates whether the observed differences in correlations are likely due to chance. The test was performed with 50,000 permutations, randomly shuffling group assignments while preserving within-group dependencies.

## Results

We first compared the three groups regarding age, cognitive ability, receptive and expressive language age, adaptive skills, and executive functions. As shown in Table 1, there were no differences in chronological age between the DD and TD-CA groups (age-matched), nor between the DD and TD-LA groups on the receptive and expressive language age. However, the TD-CA group outperformed the TD-LA and DD groups on the receptive and expressive language age. As expected, significant differences were also observed between the DD and the two control TD groups in cognitive function scores, adaptive behavior, and executive function standard scores.

Given that the study’s specific interest was to compare the communicative profiles of children with DD (the study group) to each control group, we conducted (k-1) planned comparisons.

### Comparison Between the Communicative Profiles

Significant differences emerged between children with DD and TD-CA children. In contrast, the communicative profile of children with DD closely resembled those of their TD-LA peers.

First, we compared the study group (DD) to the first control group (TD-LA). The DD and TD-LA groups did not differ significantly in the quantity of most types of communicative intents and the variety of communicative intents produced. Significant differences were observed between the groups in the frequency of certain communicative intents: social greetings and choosing, both of which were significantly more frequent in the DD group (see Table 2). Next, we analyzed the means used to convey communicative intents during the protocol. As shown in Table 3, children in the DD group used significantly more body actions and gestures compared to children in the TD-LA group.

**Table 2:** Comparison of communicative intents by group

Variable	Study group DD		DD vs. TD-LA							DD vs. TD-CA						
			Control group TD-LA		U	Z	p value	Effect size (r)	Post hoc power	Control group TD-CA		U	Z	p value	Effect size (r)	Post hoc power
Communicative intent	Median	IQR	Median	IQR						Median	IQR					
Deny or object	2	2	2	2.5	248.50	−.83	.41	0.123	0.159	2	2	209.5	−1.26	.21	0.187	0.366
Request an object or action	3	5	2	3	207.50	−1.67	.09	0.248	0.452	1	4	174	−2.01	.04	0.299	0.645
Making choices	1	0	1	0	218.50	−2.20	.03	0.326	0.709	1	0	189	−2.66	.008	0.396	0.835
Draw attention	0	0	0	0	260.50	−.97	.33	0.144	0.2	0	1	185.5	−2.14	.03	0.319	0.686
Greeting	1	2	0	0	116.00	−3.93	<.001	0.582	0.984	1	1	193.5	−1.66	.09	0.248	0.535
Comment	1	2	1	3.5	284.50	−.06	.95	0.009	0.051	4	4	85.5	−3.97	<.001	0.591	0.986
Guess	0	1	1	1	278.00	−.22	.83	0.032	0.07	2	1	123	−3.26	.001	0.485	0.958
Request for information	0	1	1	1	242.00	−1.00	.31	0.149	0.211	3	7	96.5	−3.8	<.001	0.566	0.983
Answer “wh-” questions	3	4	2	2	237.00	−1.05	.29	0.157	0.226	3	2	236	−0.635	.53	0.094	0.118
Request for clarification	0	0	0	0	276.50	−.37	.71	0.055	0.094	1	1	143.5	−3.14	<.001	0.467	0.95
Conversation	0	0	0	1	231.50	−1.55	.11	0.23	0.415	2	1	51	−5.11	<.001	0.759	0.999
Variety of CI	7	2	6	2	234.00	−1.11	.26	0.165	0.242	10	3	81.5	−4.08	<.001	0.606	0.989

*DD* developmental delay, *TD-LA* typically-developed age-equivalent language, *TD-CA*  typically-developed-chronological age, *CI* communicative intent

Key observations: 1. High power results (≥ 0.99, strong ability to detect an effect); 2. Moderate power results (0.5—0.9, reasonable ability to detect an effect); 3. Low power results (< 0.5, weak ability to detect an effect)

**Table 3:** Comparison of the means used to produce the communicative intents by group

Communicative means	Study group DD		DD vs. TD-LA							DD vs. TD-CA						
			Control group TD-LA		U	Z	p value			Control group TD-CA		U	Z			
	Median	IQR	Median	IQR				Effect size (r)	Post hoc power	Median	IQR				Effect size (r)	Post hoc power
Body action	3	5	2	3	192.5	−1.99	.047	0.281	0.607	0	1	50.5	−4.88	<.001	0.719	0.999
Facial expression	0	1	0	.50	253.5	−0.88	.38	0.124	0.162	0	1	256.5	−2.21	0.83	0.325	0.705
Gaze	0	1	0	1	276.5	−0.285	.77	0.04	0.073	0	1	205	−1.89	0.058	0.278	0.527
Vocal production	0	1	0	1	274	−0.31	.76	0.043	0.076	0	0	199	−1.68	0.093	0.247	0.447
Gestures	2	4	0	1	185.5	−2.21	.03	0.312	0.694	0	0	107	−3.84	<.001	0.564	0.976
One word phrase	6	7	5	4.5	249.5	−0.79	.43	0.111	0.148	7	3	247	−0.39	0.69	0.057	0.086
Two−word phrase	2	4	3	5	215	−1.52	.13	0.215	0.436	4	3	147.5	−2.6	0.009	0.383	0.812
Three or more−word phrase	0	1	1	5	211	−1.74	.08	0.247	0.512	16	13	43	−4.99	<.001	0.735	0.999

*GDD* global developmental delay, *TD-LA*  typically-developed age-equivalent language, *TD-CA*  typically-developed- chronological age

Comparing the study group (DD) to the second control group (TD-CA), revealed notable significant differences. Children in the DD group produced more communicative intents, such as requesting objects or actions and making choices. In contrast, the TD-CA group produced significantly more requests for clarification, requests for information, guesses, conversations, attempts to draw attention, and comments. Additionally, children in the TD-CA group demonstrated a significantly greater variety of communicative intents compared to the DD group (see Table 2). We then analyzed the means used to convey these communicative intents. As shown in Table 3, children in the DD group used body actions and gestures significantly more frequently than children in the TD-CA group. Conversely, children in the TD-CA group used two- or three-word phrases significantly more frequently than children in the DD group.

It is important to note that only four variables (i.e. deny or object, answer “wh-” questions, vocal production, and one-word phrase) didn’t reach sufficient post-hock power in both comparisons.

### Association Between the Variety of Communicative Intentions and Participant Characteristics

We examined associations between the children’s characteristics and the variety of communicative intents produced during the structured pragmatic protocol. Spearman correlation analyses were conducted for each group separately (see Table 4).

**Table 4:** Spearman correlations and permutation tests for correlation differences between children's characteristics and the variety of communicative intentions produced

	Spearman correlation coefficients Variety of communicative intents^a^						
	1 DD (*n* = 23)	2 TD-LA (*n* = 25)	3 TD-CA (*n* = 23)	*p* (TD-LA vs. DD)	95% CI (TD-LA vs. DD)	*p* (TD-CA vs. DD	95% CI (TD-CA vs. DD)
*Background measures*							
Age	.35	.38	.27	0.72	[−0.16, 0.19]	0.74	[−0.15, 0.20]
MSLE standard score	.27	−.03	−.14	0.051	[−0.42, 0.01]	0.08	[−0.41, 0.05]
MSEL language receptive^b^	.70**	.34	.15	0.006	[−0.67, −0.13]	0.009	[−0.65, −0.12]
MSEL language expressive^b^	.56**	.44*	.06	0.021	[−0.41, −0.05]	0.07	[−0.40, 0.02]
Brief-P standard score	−.23	−.59**	.045	0.014	[−0.54, −0.12]	0.012	[−0.59, −0.14]
VABS	.30	.23	.03	0.59	[−0.29, 0.14]	0.61	[−0.28, 0.17]

*GDD*  global developmental delay, *TD-LA* typically-developed age-equivalent language, *TD-CA* typically-developed- chronological age, *MSLE* Mullen Scales of Early Learning (MSEL; Mullen, 1995),*VABS* Vineland Adaptive Behavior Scales, second edition (Sparrow et al., 2005)

^a^Variety of communicative intents refers to the number of different communicative intents a child produced during the protocol (e.g., requesting, commenting, greeting), regardless of frequency

^b^Language age equivalence

*p <.05; **p <.01; ***p <.001

In the DD group, a strong positive association was found between the variety of communicative intents and language abilities. In the TD-LA group, significant correlations were observed with executive functions and expressive language age. However, in the TD-CA group, none of the examined characteristics were significantly associated with the range of communicative intents.

Table 4 shows that both receptive and expressive equivalent language ages were significantly and positively correlated with the variety of communicative intents produced by the DD group. In the TD-LA group, expressive equivalent language age was positively correlated with the variety of communicative intents, while the Brief-P executive functions score was significantly and negatively correlated with the variety of communicative intents. Overall, better language abilities were associated with producing a broader variety of communicative intents in both the TD-LA and DD groups. Additionally, in the TD-LA group, stronger executive functions were also linked to a broader variety of communicative intents. In contrast, in the TD-CA group, none of the examined characteristics were significantly associated with the variety of communicative intent produced during the protocol.

Permutation test analysis revealed significant differences in correlation coefficients between the DD and TD-LA groups concerning receptive equivalent language age, expressive equivalent language age, and executive functions. Specifically, in the DD group, the correlation between receptive and expressive equivalent language age, and the variety of communicative intent produced was significantly stronger than in the TD-LA group. However, in the TD-LA group, executive function scores (Brief-P total score) showed a significantly stronger correlation with the variety of communicative intent than in the DD group. Comparisons between the DD and TD-CA groups revealed significant differences in correlation coefficients for cognitive functioning and receptive equivalent language age.

## Discussion

The current study examined communicative intentions, and the means used to express them, collectively referred to as the communicative profiles of children with developmental delays (i.e. children with ID or GDD, including children with DS), in comparison to their TD peers matched by chronological age or language ability. Additionally, the study explored the relationship between group characteristics and the variety of communicative intentions produced. Our findings reveal both notable differences and similarities in the communicative profiles across groups.

### Communicative Profile by Group

Differences were observed in the communicative profiles between children with developmental delays and their chronologically age-matched peers. Older TD children exhibited a broader and more social-in-nature range of communicative intentions (i.e., guessing, commenting, and requesting information) and predominantly used complex verbal expressions (i.e., longer phrases) to express their intent. In contrast, children with developmental delays favored non-verbal communication means, such as body actions and gestures. However, when these children were compared with their language-age-matched TD peers, the communicative profiles appeared more similar. Both groups produced similar quantities and types of communicative intentions, except for the higher number of social greetings and choice-making produced and the use of non-verbal means (e.g., body actions, gestures) among children with developmental delays, indicating a possible compensatory strategy to mitigate verbal communication challenges.

Our findings are consistent with previous research on the production of communicative intentions in children with ID, which has shown similarities between children with ID and their TD peers matched by mental age (Abbeduto et al., 1988; Yoder & Warren, 2002). Specifically, the observation that children with developmental delays exhibit communicative profiles similar to those of TD peers with equivalent language age, yet show notable differences compared to their chronological age-matched TD peers, supports prior studies linking pragmatic abilities to cognitive and verbal skills (Gooch et al., 2016; Lee et al., 2017).

### The Association Between Communicative Intents and Participant Characteristics

The ability to produce a broader variety of communicative intentions (i.e., a greater number of different types) in both language-matched groups was associated with better verbal ability. Children with stronger verbal skills produced a wider variety of communicative intentions, aligning with established relationships between language and pragmatic ability (Matthews et al., 2018). However, among language-equivalent TD children, executive functions were also linked to the variety of communicative intentions produced. This cognitive resource, which is known to be delayed in children with intellectual disabilities (Memisevic & Sinanovic, 2014), plays a crucial role in pragmatic and communicative intention development (Cummings, 2023). Indeed, previous research has demonstrated a relationship between executive functions and pragmatic abilities in both TD children (Blain-Brière et al., 2014; Matthews et al., 2018) and school-age children with intellectual disabilities (Lee et al., 2017). Unlike previous studies, however, the association between executive functions and pragmatics in our study was found only among young TD children. This discrepancy may stem from differences in age groups, as Lee et al. (2017) examined older children and pragmatic area (Blain-Brière et al., 2014).

Taken together, our findings point to a delay in the acquisition of communicative intentions. Children with DD exhibited a communicative profile similar to their language-matched TD peers but differed from their age-matched TD peers. On the other hand, our findings also indicate that the variety of communicative intentions produced was associated with different cognitive abilities compared to both matched TD groups, suggesting potentially distinct underlying acquisition processes. Indeed, previous studies examining broader or more complex pragmatic abilities in older children with intellectual disabilities have found persistent differences—specifically, delays in production—even when controlling for mental ability (Lee et al., 2017; Smith et al., 2017).

Our findings align with the Emergentist Model of Pragmatic Ability and Disability, which suggests that pragmatic skills arise from the interplay of cognitive, linguistic, and sensory-motor systems (Perkins, 1998, 2000, 2005; Perkins, & Azios, 2024). Indeed, older TD children with more developed cognitive and linguistic resources demonstrated more advanced pragmatic behaviors. This was evident in their production of a greater number and variety of communicative intents, including higher-level functions such as requesting information or commenting, as well as their use of more complex means (e.g., longer phrases).In contrast, children with DD and their language-age-equivalent TD peers may still struggle to express communicative intentions that require more advanced cognitive and linguistic abilities reinforcing the idea that communicative intent evolves alongside cognitive and language abilities.

Moreover, for children with DD, communicative intention acquisition appears to be primarily associated with language abilities, including comprehension and expression. In younger TD-LA children, however, communicative intention production is influenced by broader cognitive resources, encompassing both expressive language skills and executive functions. This distinction suggests that children with DD primarily rely on language abilities when producing communicative intentions. Such reliance may serve as a compensatory adaptation in the early stages of communicative intention acquisition, allowing them to exhibit communicative behaviors similar to younger TD children with comparable language skills. However, given the expected developmental trajectory of TD children in acquiring communicative intentions during the preschool years, we hypothesize that overreliance on language abilities may hinder the development of more advanced communicative intentions later on. This aligns with the concept of ‘partial compensation,’ where limited cognitive resources provide short-term adaptation but may not fully support the long-term acquisition of typical abilities (Livingston & Happé, 2017). Consequently, this may lead to differences in the future acquisition of more complex communicative intentions for the DD group, but not for their language-aged TD peers, who rely on broader cognitive resources.

Another potential mechanism underlying the observed differences between children with DD and their TD peers may involve neural differences in pragmatic processing. Previous research on other neurodevelopmental populations, such as children with ASD, has revealed differences in neural mechanisms involved in processing pragmatic information, particularly in brain regions associated with social cognition and executive function (Livingston & Happé, 2017). However, these neural mechanisms have not been extensively studied in children with DD. Given that pragmatic abilities require the integration of multiple cognitive processes, differences in neural connectivity or activation patterns may contribute to the delayed or altered acquisition of communicative intentions in children with DD. Furthermore, these neural and cognitive differences may interact synergistically with environmental and familial influences. Environmental factors, such as parental communicative style, can also play a crucial role, as atypical child behaviors might shape parent–child interactions either positively (e.g., parents adopting compensatory behaviors aimed at enhancing their child’s communication) or negatively (e.g., children receiving more directive interactions) (Blacher et al., 2013; Wan et al., 2019). Thus, future research should investigate the interplay between neural mechanisms, cognitive abilities, and environmental influences to better understand pragmatic development and communicative intentions in children with DD.

Among the age-equivalent TD children, the variety of communicative intentions produced was not associated with the personal variables examined in this study, such as age, language ability, or executive functions. The mean chronological age of the age-matched TD children was four years and seven months; since most of the communicative intentions assessed in this study are typically acquired by this age (Klecan-Aker & Swank, 1988), it is possible that they had already become automatic and, therefore, did not correlate with the personal characteristics examined. Furthermore, these children more frequently produced more socially advanced communicative intentions, such as clarification, requests for information, and guessing. These intentions rely on mentalization abilities- such as understanding or inferring others’ thoughts, intentions, and knowledge. For example, clarification requires the speaker to recognize that the listener may not have understood or that they are not being understood, while requesting information depends on the realization that the other person holds knowledge that the speaker lacks. When combined, these abilities facilitate more successful, reciprocal, and adaptive communication. Therefore, it can be assumed that, for these older TD children, the ability to produce a wider variety and more advanced communicative intentions is associated with other cognitive resources, such as the Theory of Mind.

We acknowledge that our DD group was heterogeneous, as it included children diagnosed with GDD, intellectual and developmental disabilities (IDD), and DS. Although our statistical comparisons did not reveal significant group differences within the DD group, given that children with DS are known to have unique social-communicative characteristics, this heterogeneity may have influenced the findings obtained during the protocol. To address this issue, we recommend that future studies also examine communicative abilities in more specific subgroups within the DD population, allowing for a more nuanced understanding of how different developmental conditions shape communication.

The present study has several limitations. Firstly, this study utilized a methodology of direct observation of a structured protocol; perhaps examining communicative abilities in unstructured settings could provide a more comprehensive understanding of children’s communicative profiles. The child’s background variables were primarily collected through questionnaires, and no objective assessments were conducted, which may introduce bias depending on the informant. We acknowledge that the cross-sectional design of our study limits the ability to infer developmental trajectories of pragmatic language. Since our analysis was based on a single time point, it does not capture how pragmatic abilities evolve in children with developmental delays. To address this limitation, we recommend future longitudinal studies that track the development of pragmatic abilities in different settings and over time. Such research would provide a better characterization of how these abilities progress, offering deeper insights into pragmatic language development. This, in turn, could contribute to the design of more accurately tailored intervention protocols to support children with developmental delays more effectively.

The children in the study were of similar racial backgrounds; however, we acknowledge the potential influence of demographic factors, such as socioeconomic status, gender, or caregiver social-cultural characteristics, on the variety of communicative intentions produced due to caregiver communication practices (Fannin et al., 2018). Therefore, future research should explore how these sociocultural factors contribute to variations in pragmatic abilities.

The Peanut Butter Protocol we used was originally designed to elicit pragmatic behaviors in preschool children (ages 3–5); as our participants were younger, we slightly adapted the protocol. We acknowledge the importance of further validating the protocol for this age group. Lastly, Post-hoc power analysis showed that for most variables, at least one comparison yielded sufficient power, with medium or strong effect sizes. Since post-hoc power can be calculated based on either the current study’s data or previous research findings, we assumed that conclusions could be drawn for both comparisons even if adequate power was achieved in only one of them. Despite the small sample size, most variables demonstrated sufficient post-hoc power. However, four variables (i.e. deny or object, answer “wh-” questions, vocal production, and one-word phrase) did not achieve sufficient post-hoc power and also did not yield significant results. This suggests either that no meaningful difference exists for these variables or that a subtle effect may be present, requiring a larger sample size to draw more definitive conclusions.

The research findings have significant clinical implications. This study highlights the importance of strengthening verbal abilities and executive functions, as these foundational skills may contribute to pragmatic language development, particularly the production of communicative intentions. Given that children with developmental delays often struggle with these underlying abilities, interventions should be tailored to each child’s developmental stage. Initially, intervention should focus on reinforcing nonverbal communication skills (e.g., gestures) before gradually expanding verbal abilities through visual aids and modeling techniques. To support executive function development, one effective approach is the use of navigation maps, which serve as visual aids to enhance organization and planning. Additionally, play-based activities and games that target inhibition, cognitive flexibility, and working memory (Zhang et al., 2025) can further strengthen executive functions. By integrating these strategies, interventions can enhance both language and executive function skills, which, in turn, may indirectly foster the development of pragmatic communication.

## Conclusions

A comparison of the communicative profiles of children with developmental delay to those of their typically developing peers reveals delayed communicative intention acquisition. However, deeper probing of variables associated with the variety of communicative intentions produced points to possible underlying diversity (i.e., association with different cognitive resources) between children with developmental delays and TD children, particularly those matched in language abilities. Thus, our findings indicate the possibility of delays and differences in the acquisition of communicative intention acquisition in children with developmental delays.

## Supplementary Information

Below is the link to the electronic supplementary material.

Supplementary file1 (DOCX 18 KB)
