# Supplementary material for: Communicative Profiles of Children with Developmental Delay Compared to Age- or Language-Matched Typically Developing Peers
Source: J Autism Dev Disord. 2025 May 23;56(11):4227–41. doi: 10.1007/s10803-025-06882-0 (PMC13687298; doi:10.1007/s10803-025-06882-0)
Supplement: Supplementary file 1 [file 10803_2025_6882_MOESM1_ESM.docx]

| **Communicative Intent** | **Prompt for Elicitation** | **Examples of Child’s Responses** | **Means of Communication** |
| --- | --- | --- | --- |
| **Denying or objecting** | The experimenter offers the child another color but hands them a stick instead. | Child gestures "No"; child says, "It's not a crayon." | **Gesture, multi-word utterance** |
| **Requesting an object or action** | The experimenter gives the child paper and asks them to draw but does not provide a crayon. | Child says, "Crayon"; child looks at experimenter and reaches out their hand. | **Word, gesture** |
| **Making choices** | Experimenter asks, "Do you want the red or the blue crayon?" | Child names a color or reaches out for the chosen crayon. | **Verbal response, body movement** |
| **Drawing attention** | Experimenter "accidentally" drops an object on the floor. | Child points to the fallen object and looks at the experimenter. | **Gaze and pointing** |
| **Greeting and closing** | Experimenter calls the child, and child calls experimenter back using a toy telephone. | Child says "Hello" or "Bye." | **Verbal or gestural response** |
| **Commenting on an event** | Experimenter presents an everyday object in an unusual manner, awaiting child’s reaction. | Child looks at experimenter and smiles; child says, "Big." | **Gaze and smile, words** |
| **Guessing** | Experimenter asks, "What do you think is inside the box?" | Child provides an answer. | **Word**s |
| **Requesting information** | Experimenter asks child to bring the green bag, which is not in sight. | Child asks, "Where?" | **Verbal response** |
| **Answering WH-questions** | Experimenter and child play with a tickling brush. Experimenter asks, "Where should I tickle you?" | Child answers verbally or points to a body part. | **Verbal response, pointing** |
| **Requesting clarification** | Experimenter asks, "Do you want to draw with… (mumbles indistinctly)?" | Child says, "What?" | **Verbal response** |
| **Conversational turn-taking** | Experimenter engages the child in conversation. | Child responds to questions with more than a yes/no or single-word answer, initiates at least one new idea, or provides additional information, or asks a question. | **Verbal response** |

Table 1. Pragmatic Protocol (Drawing Format, Part A) adapted from the Peanut Butter Protocol (Creaghead, 1984)
